# Rational design of PD-1-CD28 immunostimulatory fusion proteins for CAR T cell therapy

**DOI:** 10.1038/s41416-023-02332-9

**Published:** 2023-07-04

**Authors:** Theo Lorenzini, Bruno L. Cadilha, Hannah Obeck, Mohamed-Reda Benmebarek, Florian Märkl, Stefanos Michaelides, Thaddäus Strzalkowski, Daria Briukhovetska, Philipp Jie Müller, Sayantan Nandi, Pia Winter, Lina Majed, Ruth Grünmeier, Matthias Seifert, Svenja Rausch, Tobias Feuchtinger, Stefan Endres, Sebastian Kobold

**Affiliations:** 1 Division of Clinical Pharmacology, Department of Medicine IV, LMU University Hospital, LMU Munich, Germany, Member of the German Center for Lung Research (DZL), Munich, Germany; 2 National Cancer Institute (NCI), Bethesda, MD USA; 3 Department of Pediatric Hematology, Oncology, Hemostaseology, and Stem Cell Transplantation, Dr. von Hauner University Children’s Hospital, LMU University Hospital, LMU, Munich, Germany; 4 German Center for Infection Research (DZIF), Munich, Germany; 5 German Center for Translational Cancer Research (DKTK), partner site Munich, Munich, Germany; 6 Einheit für Klinische Pharmakologie (EKLiP), Helmholtz Zentrum München, German Research Center for Environmental Health (HMGU), Neuherberg, Germany

**Keywords:** Cancer immunotherapy, Immunotherapy, Haematological cancer, Tumour immunology, Cancer therapy

## Abstract

**Background:**

In many situations, the therapeutic efficacy of CAR T cells is limited due to immune suppression and poor persistence. Immunostimulatory fusion protein (IFP) constructs have been advanced as a tool to convert suppressive signals into stimulation and thus promote the persistence of T cells, but no universal IFP design has been established so far. We now took advantage of a PD-1-CD28 IFP as a clinically relevant structure to define key determinants of IFP activity.

**Methods:**

We compared different PD-1-CD28 IFP variants in a human leukemia model to assess the impact of distinctive design choices on CAR T cell performance in vitro and a xenograft mouse model.

**Results:**

We observed that IFP constructs that putatively exceed the extracellular length of PD-1 induce T-cell response without CAR target recognition, rendering them unsuitable for tumour-specific therapy. IFP variants with physiological PD-1 length ameliorated CAR T cell effector function and proliferation in response to PD-L1^+^ tumour cells in vitro and prolonged survival in vivo. Transmembrane or extracellular CD28 domains were found to be replaceable by corresponding PD-1 domains for in vivo efficacy.

**Conclusion:**

PD-1-CD28 IFP constructs must mimic the physiological interaction of PD-1 with PD-L1 to retain selectivity and mediate CAR-conditional therapeutic activity.

## Background

Adoptive transfer of chimeric antigen receptor (CAR) T cells has greatly impacted the field of anti-tumour immunotherapies [1]. Anti-CD19 CAR T cells have shown unparalleled response rates in haematologic malignancies, leading to the approval for treatment of acute lymphoblastic leukemia (ALL) and advanced B-cell lymphoma [2–4]. Since then, the number of approved therapeutic CAR T cell products has steadily increased [5–7]. However, despite excellent initial response rates, remission longer than 12 months in ALL is only maintained in 40 to 60% of patients [2, 8, 9]. The mechanisms underlying these relapses can be tumour-intrinsic (e.g., loss of target antigen) but are mainly attributed to loss of function, poor expansion, and short persistence of active CAR T cells [2, 10]. This dysfunctional state, also known as T cell exhaustion, has been identified as a major contributor to the failure of anti-tumour immune response and has been thoroughly investigated [11]. In particular, this phenomenon is characterised by the expression of inhibitory receptors on T cells, such as programmed cell death protein 1 (PD-1), which interacts with its ligands programmed death-ligand 1 (PD-L1) and PD-L2. PD-1 expression correlates with poor clinical outcomes in cancer patients as tumour cells can exploit this interaction to dampen T cell function and evade anti-tumour immune response [12]. Therefore, immune checkpoint blockade (ICB) targeting the PD-1-PD-L1 axis has emerged as a strategy for overcoming T cell exhaustion and enhancing anti-tumour immunity with tremendous clinical success [12, 13]. PD-1-PD-L1 ICB also became an evident combination partner for anti-CD19 CAR T cell therapy: several clinical reports found that in CAR T cell-treated patients with refractory or relapsed disease, PD-1-targeted ICB was able to restore the initial response by boosting CAR T cell activation and proliferation, leading to complete disease remission in some individuals [14–16].

However, the application of anti-CD19-CAR T cells alone bears a significant risk of causing immune-related adverse events [2, 8, 9]. Blocking the PD-1-PD-L1 axis through systemic use of antibodies such as nivolumab or pembrolizumab also comes with a relevant immune-related side effect profile [13, 17, 18]. Thus, a combination with CAR T cell therapy can significantly increase the threat of exacerbating side effects. A selective blockade of the PD-1-PD-L1 axis solely on the infused CAR T cells could allow for more selective activation and a more contained spectrum of immune-related side effects.

A known and elegant approach that has been applied to stem the disadvantages of systemic targeting of the PD-1-PD-L1 axis relies on the conversion of PD-1-mediated suppression into a CD28-mediated costimulatory signal by introducing immunostimulatory fusion protein (IFP) constructs into adoptively transferred cells. These fusion proteins combine the extracellular domain of PD-1 with the intracellular domain of CD28 and have been previously introduced by us and others [19–21]. Within the context of adoptive T-cell therapy, this novel approach has been successfully validated in various tumour models such as ALL, non-Hodgkin lymphoma, and solid tumours [19, 22–25]. Owing to their sole expression on adoptively transferred tumour-specific T cells, IFPs effectively bypass the disadvantages of systemic targeting of immunosuppressive axes and are granted the potential to substantially reduce immune-related adverse effects [19]. These advantages position IFPs as an attractive strategy to overcome immunosuppression in tumour-specific T cells.

Recently, a novel IFP designed to convert an inhibitory CD200R stimulus into a CD28 costimulus demonstrated how tuning different IFP domains can drastically alter the therapeutic effects in a preclinical model of acute myeloid leukemia (AML) [26]. For instance, it was postulated that the inclusion of a dimerisation-prone motif from the CD28 extracellular domain results in improved therapeutic efficacy and outcompetes other designs. We questioned if this observation would stand across different IFP families, targeting other molecules such as PD-1. For IFPs currently under development, the confirmation of these observations would prompt implementation into upcoming clinical protocols, as there is no consensus on a universal IFP architecture that could be applied to all inhibitory receptor families. Within this study, we thus elucidate the properties of the underlying domains and their significance by experimentally comparing different structural variants of a human PD-1-CD28 IFP on anti-CD19 CAR T cells, aiming to achieve a functionally enhanced, PD-L1 resistant therapeutic product for leukemic cells.

## Methods

### Animal experimentation

NSG (NOD.Cg-Prkdc^scid^ Il2rg^tm1WjI^/SzJ) mice were purchased from Charles River (Sulzfeld, Germany) or Janvier (St Berthevin, France). Animals were housed in specific pathogen-free facilities. All animal studies were approved by the local regulatory office on animal experimentation (*Regierung von Oberbayern*). In accordance with the animal experiment application, the health status of mice was checked at least three times per week. For ethical reasons and imposed by animal care regulations, endpoints of survival studies were defined as weight loss above 20% or other clinical signs suggestive of advanced leukemia in mice, namely irresponsiveness to stimuli, abnormal posture, and hind limb paralysis. These endpoints were registered by blinded observers and used as surrogate endpoints for survival and are used as such throughout the study.

### In vivo treatments and tumour growth studies

Tumour cells were injected intravenously in 100 µl PBS into the tail vein of mice. Tumour burden was measured through bio-luminescence signal using an IVIS Lumina X5 from Perkin-Elmer (Waltham, MA, USA) at a frequency indicated in the figure legends. Mice were injected with 100 µl of XenoLight D-Luciferin potassium salt, (Perkin Elmer, USA) 10 min prior to imaging according to the manufacturer’s instructions. The Living Image Software 4.7.2 (Perkin-Elmer) was used for the analysis of acquired images. Luminescence is depicted as radiance (photons/sec/cm^2^/sr). For treatment with adoptively transferred T cells, 10^7^ T cells were injected intravenously in 100 µl PBS. Mice were allocated randomly to treatment groups by the blinded observer.

### Preparation of single-cell suspensions, antibody staining, and flow cytometry

Dead cells were stained using eFluor 780 Fixable Viability Dye (eBioscience, Thermofisher), followed by blocking of Fc receptors with TruStain FcX (BioLegend, San Diego, CA, USA). Cell surface proteins of human T cells were stained with the following antibodies: anti-c-myc (SH1–26E7.1.3, Miltenyi Biotec, Germany) to determine CAR expression, anti-human CD279/PD-1 (EH12.2H7), anti-human CD3 (OKT3), anti-human CD8a (HIT8a), anti-human CD4 (OKT4), anti-human CD366/TIM-3 (F38-2E2), anti-human CD223/LAG-3 (11C3C65) and anti-human CD25 (M-A251), all from BioLegend. Cells were analyzed on a LSRFortessa flow cytometer (BD Biosciences, Germany) and data were analyzed with FlowJo software version 10.3.

### Generation of PD-1-CD28 fusion constructs

All DNA constructs were generated by overlap extension PCR and recombinant expression cloning into the retroviral pMP71 vector using standard molecular cloning protocols [27] as follows: the PTM construct (short for PD-1 transmembrane domain) consists of human PD-1 (Uniprot Entry Q15116, amino acids (aa) 1–191) and human CD28 (Uniprot Entry P10747, aa 180–220), the CTM construct (short for CD28 transmembrane domain) consists of PD-1 (aa 1–170) and CD28 (aa 153–220), the CTM + 41EC construct (short for CD28 transmembrane domain and 41 aa from the extracellular portion) consists of PD-1 (aa 1–170) and CD28 (aa 112–220), the CTM + 39EC construct (short for CD28 transmembrane domain and 39 aa from the extracellular portion) consists of PD-1 (aa 1–170) and CD28 (aa 114–220), the CTM + 12EC construct (short for CD28 transmembrane domain and 12 aa from the extracellular portion) consists of PD-1 (aa 1–170) and CD28 (aa 141–220) and the CTMΔ12EC construct (short for CD28 transmembrane domain and 12 aa from the extracellular portion of CD28, while removing 12 aa from the membrane-proximal PD-1 portion) consists of PD-1 (aa 1–158) and CD28 (aa 141–220).

### Cell line generation, culture, and validation

The human Nalm-6-PD-L1 tumour cell line was generated as previously described from wildtype Nalm-6 cells by cloning the human PD-L1/CD274 gene into a retroviral pMP71 vector (kindly provided by C. Baum, Hannover) and subsequent transduction and flow cytometry-assisted bulk sorting of positive cells [25]. Tumour cells were cultured in RPMI 1640 (Sigma-Aldrich, Saint Louis, MO, USA) supplemented with 10% heat-inactivated foetal calf serum (FCS), 100 IU/ml penicillin, 100 µg/ml streptomycin, and 2 mM L-glutamine. 293Vec-Galv, and 293Vec-RD114 were a kind gift from Manuel Caruso, Québec, Canada. Retroviral pMP71 vectors carrying the sequence of the relevant receptor were stably introduced in packaging cell lines to generate producer cell lines of the desired constructs [28]. The following producer cell lines were generated: 293Vec-RD114 for PTM, CTM, CTM + 12, CTM + 39, CTM + 41, CTMΔ12EC, and anti-CD19-CAR. After written informed consent in accordance with the Declaration of Helsinki and approval by the Institutional Review Board of the Ludwig-Maximilians-Universität (Munich, Germany), human T cells were isolated from healthy donors, transduced and cultured according to previously described protocols [29]. All cell lines used in experiments were regularly checked for mycoplasma species with the commercial testing kit MycoAlert (Lonza, Basel, Switzerland). Short tandem repeats DNA profiling analysis was conducted in-house to authenticate human tumour cell lines. Cells were not cultured for a period longer than eight weeks.

### Proliferation assays

The proliferation of transduced T cells was measured at indicated time points after coculture with tumour cells using a flow cytometry-based CellTrace Violet Cell Proliferation Kit from Invitrogen (Waltham, MA, USA) according to the manufacturer’s protocol.

### Cytotoxicity assays and cytokine protein level quantification

T cells were incubated with Nalm-6 tumour cells at indicated effector-to-target ratios. Following 24 to 48 h of coculture, the supernatant was removed to quantify the indicated human cytokine concentration by ELISA (BD Biosciences, Franklin Lakes, NJ, USA). Subsequently, the Bio-Glo Luciferase Assay System (Promega, Madison, WI, USA) was used according to the manufacturer’s protocol to determine tumour cell lysis.

### Confocal microscopy

Blinded confocal imaging and conjugate quantification were carried out as previously described [30]. Cells were cocultured in a V-well plate before transfer to a poly-L-lysine-coated slide, on which the cells were allowed to adhere for 30 min before fixation and permeabilization. Leica TCS SP5 confocal system with an HCX PL APO CS 63x/1.4 oil objective was used for image acquisition on Leica application suite v2.7.3.9723. Following the selection of 3 or more representative areas on each slide, T cells in or out of conjugate were quantified. F-actin and its polarisation to the immune synapse, or lack thereof, was noted to determine functional synapses.

### Western blot

Jurkat E6.1 (ECACC 88042803) tumour cells were retrovirally transduced with IFP constructs and bulk sorted for positive cells. The cells were then lysed with RIPA cell lysis buffer and centrifuged at 15,000 g for 10 min. The resulting supernatant was used for total protein estimation using the Bradford assay. After protein quantification, the samples were denatured using Laemmli buffer at 95 °C for 5 min. For reducing conditions, β-mercaptoethanol was added to the Laemmli buffer in a concentration of 1:10. The samples were then subjected to sodium dodecyl sulfate-polyacrylamide gel electrophoresis (SDS-PAGE). After separating the proteins, they were transferred from the gel to a polyvinylidene difluoride membrane using the wet transfer system. The membrane was blocked with 5% bovine serum albumin for 1 h and probed with a primary antibody (anti-human CD279, clone NAT105, BioLegend) overnight. On the next day, the membrane was washed three times with 1X TBST (Tris-buffered saline with Tween) for 10 min each. Thereafter, the membrane was probed with a secondary antibody (anti-mouse IgG, HRP-linked antibody, Cell Signalling) for 1 h and washed three times with 1X TBST for 10 min each time. Following this procedure, the membrane was prepared for chemiluminescence detection.

### Statistical analysis

Statistical analysis was performed with GraphPad Prism 9 software. Data are shown as mean and error bars represent standard deviation. Statistical analysis was performed as indicated in the figure legends; non-significant differences are not depicted in the figures. *p* < 0.05 was considered statistically significant and represented as *<0.05, **<0.01, and ***<0.001. No statistical methods were used to predetermine the sample size.

## Results

### Rational design and function of PD-1-CD28 IFP variants

**Fig. 1:**
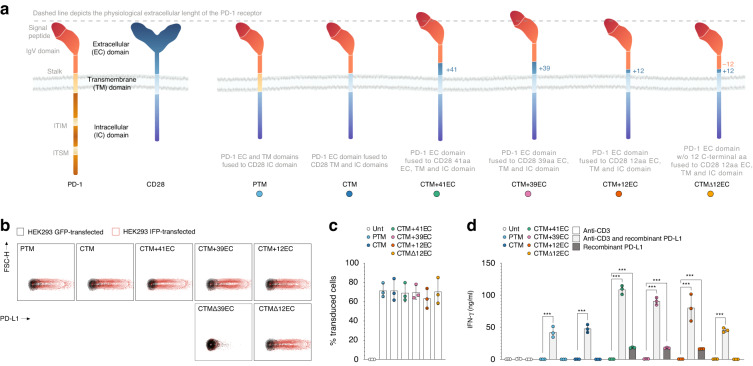
Rational design of immunostimulatory fusion proteins (IFP) based on extracellular domains. **a** Schematic representation of the original and fusion receptors employed in this study. From left to right: PD-1, CD28, PTM (short for PD-1 transmembrane domain), CTM (short for CD28 transmembrane domain), CTM + 41EC (short for CD28 transmembrane domain and 41 amino acids (aa) from the extracellular [EC] portion of CD28), CTM + 39EC (short for CD28 transmembrane domain and 39 aa from the EC portion of CD28), CTM + 12EC (short for CD28 transmembrane domain and 12 aa from the EC portion of CD28) and CTMΔ12EC (short for CD28 transmembrane domain and 12aa from the EC portion of CD28; additionally, 12 aa were removed from the membrane-proximal part of the PD-1 portion). The putative extracellular length of PD-1 is indicated by the dashed line and CTM + 41EC, CTM + 39EC, and CTM + 12EC constructs are predicted to exceed this length. **b** PD-L1 binding of HEK293 cells after plasmid transfection, detected through flow cytometry after incubation with recombinant Human PD-L1/B7-H1 Fc Chimera Protein (R&D Systems) and subsequent IgG labelling (Zenon™ Human IgG Labelling Kit, R-Phycoerythrin, Invitrogen). The experiment depicted is representative of 3 independent assays. **c** Transduction efficiency of primary human T cells from healthy donors quantified by flow cytometry through staining with a human PD-1 antibody. Three independent experiments were pooled together; each was performed with a different donor. **d** Stimulation assay of IFP-transduced human T cells with anti-CD3 (100 ng/ml) and recombinant human PD-L1 (5 µg/ml). IFN-γ concentration in the supernatant was measured as a surrogate cytokine for T-cell activation. The experiment depicted is representative of 3 independent assays with different donors. *P* values for (**d**) are based on a two-tailed unpaired *t*-test.

To determine the optimal prerequisites for the PD-1-CD28 IFP, we fused different variants of the extracellular domain of PD-1 to the intracellular domain of CD28, creating multiple variants in the protein length of the PD-1-CD28 IFP (Fig. 1a). The first two constructs consist of a physiological extracellular PD-1 domain, with the difference being in the transmembrane portion: PD-1 transmembrane domain (PTM) for the first construct and CD28 transmembrane domain (CTM) for the second. As observed in an IFP consisting of CD200R-CD28 [26], extending the CD28 transmembrane domain into the extracellular space incorporates a cysteine proximal to the cell membrane, allowing for enhanced CD28 homodimerization and thus presumably improving therapeutic efficacy (CTM + 12EC) [26, 31]. To account for the additional 12 amino acids used to enhance the dimerisation of our CTM + 12EC IFP, we generated an additional control construct where the 12 amino acids from the stalk domain of PD-1 proximal to the cell membrane were removed to restore physiological extracellular length (CTMΔ12EC). SDS-PAGE confirmed that among the constructs with physiological extracellular length (PTM, CTM, and CTMΔ12EC), only CTMΔ12EC promoted dimerisation and was reversed to its monomeric state under reducing conditions (Supplementary Fig. 1A) [31, 32].

We investigated additional IFP configurations by incorporating larger portions of the extracellular CD28 domain and added 39 (CTM + 39EC) and 41 (CTM + 41EC) amino acids, to respectively include a CD28 CAR spacer domain (well-established in conventional CAR construct design) [33] as well as the next cysteine in the CD28 extracellular portion distal to the membrane. CTM + 12EC, CTM + 39EC, and CTM + 41EC are thought to exceed the physiological extracellular length of PD-1 and thus might impede optimal receptor entry into the immunological synapse and its subsequent function therein [26].

To evaluate the expression of these different PD-1-CD28 IFP constructs, we transfected HEK293 cells with all six constructs and proved that they are capable of binding PD-L1 (Fig. 1b). Similar to CTMΔ12EC, we tried to restore the physiological length of CTM + 39EC by removing 39 amino acids from the PD-1 domain and created a new construct, CTMΔ39EC. However, the CTMΔ39EC IFP could not be detected by anti-human PD-1 antibodies (Supplementary Fig. 1B), nor did it bind PD-L1 (Fig. 1b). As a consequence of shortening the PD-1 stalk by 39 amino acids, we had inevitably removed two nonpolar residues in the PD-1 domain (Ala132 and Ile134) that have been described to constitute the hydrophobic core, which is essential for the interaction with PD-L1 [34]. We thus concluded that the CTM + 39EC and the even longer CTM + 41EC could not be shortened similarly as the CTMΔ12EC construct, as it would eliminate their ability to bind PD-L1 and disqualified their shortened versions from being used in the following experiments.

Our PD-1-CD28 IFP constructs yielded comparable transduction efficiencies among the different constructs in primary human T cells (Fig. 1c). To create a set-up with precise amounts of PD-L1 exposure while still triggering T cell activation through the T cell receptor (TCR), we used agonistic anti-human CD3 antibodies in the presence or absence of recombinant PD-L1. Upon stimulation with a CD3 antibody and recombinant PD-L1, all PD-1-CD28 IFP-transduced T cells triggered a measurable IFN-γ secretion, confirming their functionality and synergistic action with TCR signalling (Fig. 1d). Interestingly, and in contrast to the other constructs, all PD-1-CD28 IFP constructs whose extracellular length exceeded the putative physiological PD-1 length (CTM + 12EC, CTM + 39EC, and CTM + 41EC) showed substantial IFN-γ release upon contact with PD-L1 in the absence of TCR signalling. This suggests TCR-independent T cell activation of these constructs, triggered by the exposure to PD-L1.

### PD-1-CD28 IFPs complement the anti-CD19 CAR activity in vitro, whereas excessive extracellular length induces CAR-independent T-cell activation

**Fig. 2:**
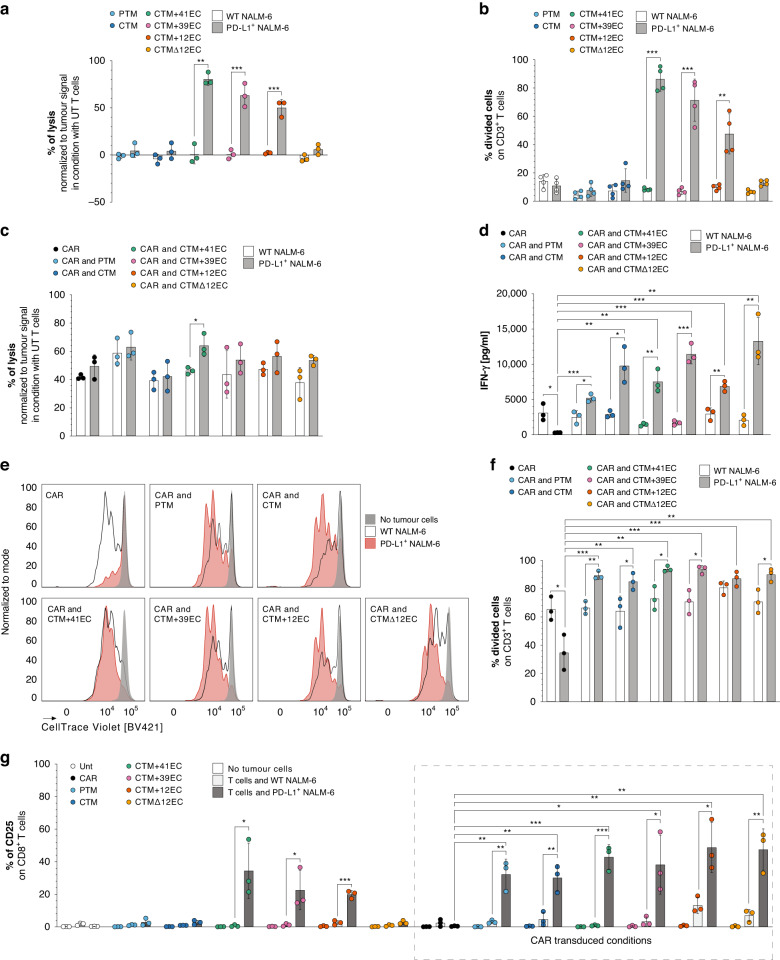
IFP components determine stand-alone or add-on function to the anti-CD19 CAR. **a** Coculture assay of IFP-transduced T cells with luciferase positive Nalm-6 tumour cell lines (WT and PD-L1^+^) after 24 h with an effector to target (E/T) ratio of 1/1. Results from luminescence readout presented as % of lysis after normalisation to the tumour signal of the coculture with untransduced (UT) T cells. Each dot is representative of a different donor, analyzed in independent experiments. Data pooled together from 3 independent experiments. **b** Percentage of proliferated IFP-transduced T cells after 72 h of coculture with Nalm-6 tumour cell lines with an E/T ratio of 1/1. Each dot is representative of a different donor, analyzed in independent experiments. Data pooled together from 4 independent experiments. **c** Results from luminescence readout presented as % of lysis after normalisation to the tumour signal of the coculture with untransduced T cells. CAR- and IFP-transduced T cells were cocultured with Nalm-6 tumour cell lines for 48 h (E/T ratio 1/10). Each dot is representative of a different donor, analyzed in independent experiments. Data pooled together from 3 independent experiments. **d** Results of ELISA for IFN-γ as a surrogate cytokine for T cell activation. Supernatants were harvested from a coculture assay after 48 h of CAR- and IFP-transduced T cells with Nalm-6 tumour cells. The experiment depicted is representative of 3 independent assays with different donors. **e**, **f** Histogram plots and bar graph with absolute quantification, respectively, of CAR- and IFP-transduced T cell proliferation after 72 h of coculture with Nalm-6 tumour cell lines. Results in (**e**) are from one donor representative of three different donors analyzed in independent experiments that are quantified and depicted in each dot in (**f**). **g** Flow cytometry analysis of CD25 % of positive cells on CD8^+^ T cells after 48 h of coculture with Nalm-6 tumour cells (E/T ratio 1/1). Each dot is representative of a different donor, analyzed in independent experiments. Data pooled together from 3 independent experiments. *P* values for (**a**–**d**, **f** and **g**) are based on a two-tailed unpaired *t*-test.

To determine if the activation of the CTM + 12EC, CTM + 39EC, and CTM + 41EC constructs seen in the presence of recombinant PD-L1 could be reiterated with PD-L1^+^ tumour cells, we cocultured transduced T cells with either wild-type (WT) or PD-L1^+^ Nalm-6 tumour cells that bear an identical expression of CD19 (Supplementary Fig. 2A,  B). As a result, T cells equipped with CTM + 12EC, CTM + 39EC, and CTM + 41EC constructs were able to specifically kill PD-L1-expressing tumour cells but not WT tumour cells. In contrast, T cells with PTM, CTM, and CTMΔ12EC constructs were unable to lyse tumour cells, irrespective of their PD-L1 status (Fig. 2a). As a further sign of PD-L1-induced activation, CTM + 12EC, CTM + 39EC, and CTM + 41EC constructs also promoted proliferation in transduced T cells when exposed to PD-L1^+^ tumour cells (Fig. 2b). Similar to before, increased proliferation was not seen in T cells transduced with PTM, CTM, or CTMΔ12EC.

To further study differences in the anti-tumour killing capacity of the generated IFP constructs, we equipped PD-1-CD28 IFP-transduced T cells with a first-generation anti-CD19 CAR containing an intracellular CD3ζ domain. The in vitro killing capacity of the T cells did not change for most PD-1-CD28 IFP- and CAR-transduced conditions (Fig. 2c), regardless of PD-L1 expression and its subsequent conversion into a CD28 stimulus, confirming that antigen-mediated cytolytic effects are independent of CD28 costimulation [35, 36]. While in vitro tumour lysis of PD-L1^+^ tumour cells was not improved by the addition of an IFP to the CAR T cells, their cytokine secretion in terms of IFN-γ (Fig. 2d), IL-2 (Supplementary Fig. 2C) and Granzyme B (Supplementary Fig. 2D) concentration in the coculture supernatant was higher than control T cells that were transduced with CAR only. Analogously, CD107a expression on CAR- and IFP-transduced T cells in coculture with PD-L1^+^ Nalm-6 were higher than on control CAR T cells (Supplementary Fig. 2E). The addition of an IFP did not only protect CAR T cells from reduced proliferation upon contact with PD-L1^+^ Nalm-6, due to the IFP-mediated conversion into a CD28 stimulus, they also exhibited significantly higher proliferative rates than control CAR T cells (Fig. 2e, f). Blocking the PD-1-PD-L1 axis conventionally by adding a PD-1 antibody to the culture had similar mechanistic effects on CAR T cells as the IFP (Supplementary Fig. 2F, G). Furthermore, CTM + 12EC, CTM + 39EC, and CTM + 41EC IFP constructs induced higher expression of activation and exhaustion markers such as CD25, TIM-3, and LAG-3 upon contact with PD-L1^+^ Nalm-6. CAR- and IFP-transduced T cells also exhibited higher CD25 and LAG-3 expression levels than control CAR T cells when cocultured with PD-L1^+^ Nalm-6 (Fig. 2g and Supplementary Fig. 2H).

These results demonstrate that IFP constructs that exceed the physiological extracellular length (CTM + 12EC, CTM + 39EC, and CTM + 41EC) induce CAR-independent T cell activation (Fig. 2g), proliferation (Fig. 2b) and tumour lysis (Fig. 2a) upon coculture with PD-L1^+^ Nalm-6 tumour cells in vitro. This effect was not promoted by IFP constructs with physiological extracellular length (PTM, CTM, or CTMΔ12EC). As intended by design, the IFP should depend on target antigen recognition of the anti-CD19 CAR and provide the additional CD28 stimulus to the T cell only upon exposure to PD-L1. Although all constructs were able to ameliorate CAR T cell effector function and proliferation in response to PD-L1^+^ tumour cells and showcase the potential of IFPs as a novel approach for interrupting the PD-1-PD-L1 axis, only PTM, CTM, and CTMΔ12EC IFPs complement the CAR without the adverse potential of inducing a CAR-independent T cell response.

### Impact of PD-1-CD28 IFP variants on immunological synapse formation

**Fig. 3:**
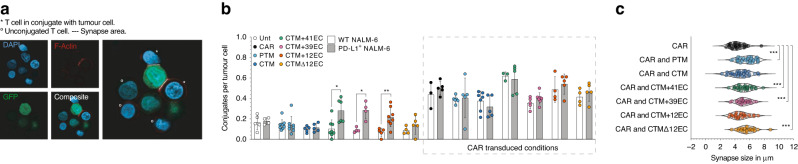
Influence of IFP design on immunological synapse formation. **a** IFP-transduced T cells and CAR- and IFP-transduced T cells were cocultured with WT and PD-L1^+^ Nalm-6 tumour cells, and functional conjugate formation was analyzed. **b** T cells in functional conjugates with tumour cells expressed by F-Actin (AF647) polarisation have been quantified in 3 to 9 independent samples per condition, the experiment is representative of 2 independent repetitions with different donors. **c** T cells in functional conjugation had their synapse area measured [µm], each dot depicts one T cell in conjugate, representative of 2 experiments with different donors. *P* values for (**b** and **c**) are based on a two-tailed unpaired *t*-test. Statistical analysis in (**c**) between CAR PTM and CAR CTM + 41EC, CAR PTM and CAR CTM + 39EC, CAR PTM and CAR CTMΔ12EC resulted in non-significant differences.

To further understand the impact of the extracellular length and structure of the PD-1-CD28 IFP and the resulting impact on immunological synapse formation, we next set out to image said synapses between transduced T cells and tumour cells (Fig. 3a). In line with our previous observations, IFP-only transduced T cells with CTM + 12EC, CTM + 39EC, and CTM + 41EC constructs formed more conjugates with PD-L1^+^ than with WT tumour cells, whereas PTM, CTM, and CTMΔ12EC constructs did not engage tumour cells in the absence of the CAR (Fig. 3b). Combination of CAR- and IFP-transduced T cell variants did not show relevant differences in the overall number of conjugates that were formed with the tumour cells (Fig. 3b). For these conjugates however, the combination of the anti-CD19 CAR with PTM, CTM + 41EC, CTM + 39EC, and CTMΔ12EC IFPs led to increased contact area of the T cell to tumour cell membrane when compared to CAR only-transduced T cells (Fig. 3c), a finding that has been associated with better T cell response [37, 38]. While CAR T cells with CTM + 41EC and CTM + 39EC IFP constructs also exhibited increased contact area, these constructs orchestrate full CAR-independent and PD-L1-mediated tumour cell engagement (Figs. 3b and  2a, b, g). Therefore, only the IFPs with physiological extracellular length, in this case, PTM and CTMΔ12EC, would promote significantly increased contact area without inducing full T cell activation in the absence of the CAR.

### PD-1-CD28 IFPs with physiological PD-1 length improve leukemic clearance and survival in vivo

We next assessed the impact of these different PD-1-CD28 IFP constructs on CAR T cell therapy in vivo. Since all constructs with excessive extracellular length (CTM + 12EC, CTM + 39EC, and CTM + 41EC) demonstrated similar CAR-independent and PD-L1-induced activation, we selected CTM + 12EC as the shortest of these constructs for testing. Based on the in vitro experiments herein shown, the IFP constructs with physiological extracellular PD-1 length (PTM, CTM, and CTMΔ12EC) enhanced T cell activation, proliferation, and cytolytic capabilities upon contact with PD-L1^+^ tumour cells. For further analysis, we thus included the PTM construct, which has previously been described to outperform the CTM construct [19], and the CTMΔ12EC construct, predicted to induce the most effective costimulation [26], which could account for the higher cytokine secretion in vitro than PTM (Fig. 2d, Supplementary Fig. 2C,  D). PTM and CTMΔ12EC also exhibited an increased synapse contact area with PD-L1^+^ Nalm-6 tumour cells when added to the CAR T cells (Fig. 3c).

**Fig. 4:**
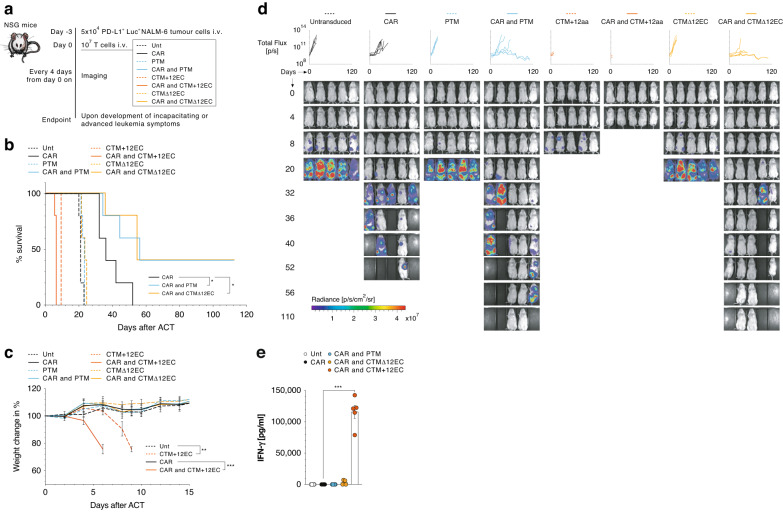
In vivo performance of CAR T cells with or without IFP constructs. **a** Experimental layout of the Nalm-6 xenograft tumour model. Mice were implanted with 5 × 10^4^ PD-L1^+^ Luc^+^ Nalm-6 tumour cells intravenously and three days after treated with a single injection of 10^7^ Untransduced, CAR-, PTM-, CAR- and PTM-, CTM + 12EC-, CAR- and CTM + 12EC-, CTMΔ12EC-, CAR- and CTMΔ12EC-transduced T cells (*n* = 5 mice per group, representative of 2 independent experiments with different donors). Mice were imaged for luminescence signals every four days. **b** Percentage survival readout. **c** Weight change in % of mice from different treatment groups. The weight for each day is normalised to the initial value on day 0. **d** Representative in vivo imaging data displaying luminescent signal in radiance for all individual mice from treatment day onwards. **e** IFN-γ levels in the serum of mice treated with anti-CD19 CAR as control and in combination with IFPs. Blood was collected on day five after ACT. Differences between relevant groups for (**b**) were calculated using Log-rank (Mantel-Cox) test. *P* values for pertinent groups of (**c**) are based on two-way ANOVA followed by Dunnett’s multiple comparisons tests. *P* values for (**e**) are based on a two-tailed unpaired *t*-test.

To test our IFP constructs, we took advantage of a luciferase positive PD-L1^+^ Nalm-6 xenograft tumour model resistant to anti-CD19 CAR T cell therapy [25]. Tumour cells were injected into NOD.Cg-Prkdc^scid^ Il2rg^tm1WjI^/SzJ (NSG) mice and treatment with transduced T cells followed three days later, while bioluminescence imaging was performed regularly to assess the tumour burden of mice (Fig. 4a). Starting four days after T cell treatment with CTM + 12EC IFP, animals began to show side effects, exhibiting reduced overall activity and rapid weight loss, regardless of additional transduction with the CAR and were removed from the experiment prematurely (Fig. 4b,  c). These mice reached predefined endpoint criteria before developing consistent tumour signals and before the untransduced T cell-treated control group mice (Fig. 4b, d). Furthermore, on day five after ACT, while none of the mice treated with CAR T cells displayed a tumour signal (Fig. 4d), IFN-γ levels in the serum spiked in all mice within the CAR and CTM + 12EC group, as a functional corroboration of the observed clinical phenotype in these mice (Fig. 4e).

T-cell treatment groups with physiological length-spanning IFPs (PTM or CTMΔ12EC) showed no difference in weight change, tumour burden, or survival compared to the untransduced group (Fig. 4b, c, d), confirming their selective function and lack of CAR-independent, PD-L1-driven T cell activation. Combining the PTM- or CTMΔ12EC IFPs with an anti-CD19 CAR resulted in improved tumour control and clearance, as evidenced by bioluminescence imaging, and led to significantly improved survival compared to treatment with CAR only (Fig. 4b, d).

## Discussion

Immune suppression is an essential tumour escape mechanism to CAR T cell therapy, which functionally leads to T cell exhaustion and disease progression [39]. IFP constructs have been proposed as a powerful strategy to overcome T cell suppression. Through the conversion of deleterious signals into proliferation and cytotoxicity, T cells equipped with an IFP can effectively drive disease control [19, 23–25]. Clinical observations confirm that leveraging the PD-1-PD-L1 axis with antibodies upon CD19-directed CAR T cell therapy improves its activity and persistence [14–16]. This warrants the investigation of novel approaches to overcome PD-1-PD-L1-mediated suppression while selectively improving CAR T cell activation and reducing systemic side effects of antibody-mediated ICB. Beyond IFP constructs, these are: local disruption of the PD-1 signalling through systemic or targeted delivery of a PD-1 blocking scFv [40], knocking out the *PDCD1* gene on CAR T cells [41] or using a dominant-negative receptor (DNR) to shield the T cell from the inhibitory signal [42]. While the experimental comparison of these approaches is beyond the goals of this study, these different strategies have inherent advantages and disadvantages due to their different mechanisms of action. Systemic deliveries of antibodies to block PD-1 or PD-L1 are approaches that can easily be reverted through therapeutic interruption of antibody administration. This is necessary for the instances when immune-related adverse effects become overwhelming following ICB therapy [13]. However, treatment discontinuation is likely insufficient for high-grade immune-related adverse effects to resolve, with patients often requiring additional glucocorticoid immunosuppression [43]. *PDCD1* knock-out approaches are elegant forms of local disruption currently being tested in clinical trials [44]. The clinical impact of this strategy is still to be published. Nevertheless, the selectivity of the approach heralds the potential to improve systemic safety but comes with the unaddressed concern of T cell editing side effects, including malignant transformation [41, 45]. Furthermore, such approaches must rely on additional engineering methods beyond standard viral infections, which come with potentially higher costs for therapy manufacturing, higher manufacture times, and increased risks of therapy manufacturing failure [46], whereas the compact DNA size of IFPs enables these receptors to be co-delivered with CAR DNA in one single viral vector without any need for extra genetical manipulation.

DNR constructs are the strategy that can most closely be compared to the concept of IFPs. A DNR will also specifically be present in adoptively transferred T cells, thus restricting the effect of inhibitory signals to T cells of known anti-tumour specificity. As DNR and IFP constructs are inserted in therapeutic T cells through genetic engineering, they could be integrated into genome regions with circumstantial reported risks of gene disruption [47]. With several genetic products currently approved for clinical use, protocols that rely on T-cell transduction are more mature than approaches that rely on gene editing. A DNR, however, differs from an IFP in its capacity to increment the function of an engineered T cell: It is built to bind its ligand and draw it from the tumour microenvironment, preventing deleterious effects [48]. In contrast, an IFP is built to harness the interaction of an immunosuppressive molecule and convert it into a stimulatory signal for the T cell. Naturally, the IFP also partly functions as a DNR as it scavenges for inhibitory molecules and thereby prevents their binding to intrinsically expressed receptors (e.g., PD-1 on T cells). To prove that the effect of the IFP does not only depend on the sequestration of inhibitory molecules, we and others have previously removed intracellular CD28 signalling motifs from the IFP and found that the additional conversion into a stimulatory signal is needed for the advantageous effects, both in vitro and in vivo [19, 22].

Notably, there has yet to be a consensus defining the best IFP structure for optimised synergy with the primary targeting receptor. Such a universal design would permit the rapid selection and implementation of IFPs for different inhibitory receptor families and likely accelerate its clinical implementation. Multiple IFP designs with different extracellular and intracellular portions have been tested in vitro and in vivo with varying degrees of efficiency. Examples include but are not limited to CTLA-4-CD28, PD-1-CD28, CD200R-CD28, CD40L-CD28, or Fas-4-1BB [26, 49–51]. The ever-slightly different molecular structure of proposed constructs highlights the need to understand the determinants of IFP function. Recently, Oda and colleagues compared different CD200R-CD28 IFP variants and determined two factors to be critical for optimal function: the incorporation of a membrane-proximal cysteine of CD28 to allow for enhanced homodimerization and access to the immune synapse by restoring the physiological extracellular length of the IFP [26]. Interestingly, this concept has already been proposed by Prosser and colleagues before; it was the initial design of a PD-1-CD28 IFP and proved to enhance T cell proliferation and cytokine secretion in vitro [21]. This variant, which is comparable to our CTMΔ12EC, has subsequently been used by Liu and colleagues to enhance CAR T cell therapy with promising effects on human xenograft tumours in mice [22] and has recently been combined with anti-CD19 CAR T cells for the treatment of large B-cell lymphoma in a limited number of patients. Out of 17 patients, the treatment yielded clinical response in 10 patients, of which 7 showed complete remission. Additionally, no adverse events (cytokine-release syndrome and severe neurological toxicity) were reported [52]. While further trials are needed to establish conclusive evidence, these results demonstrate the therapeutic potential and safety of this combinatorial approach.

A different human PD-1-CD28 IFP with a full extracellular PD-1 domain similar to our CTM design was proposed by Ankri et al. and indicated that the cysteine-mediated dimerisation was not needed for PD-1-CD28 IFP function [20]. In fact, Schlenker and colleagues compared these two previously mentioned constructs and observed that they both improve functional T cell response, with no significant difference between the constructs [53]. We previously created a murine panel of different PD-1-CD28 IFP variants and proved that a construct with extracellular and transmembrane PD-1 domain (PTM) outperforms the other two designs [19]. This design, however, has not been included in studies with human PD-1-CD28 IFPs. For this reason, we set out to create a human panel of different variants and investigate the impact of the PD-1 domains, overall length, and cysteine-mediated dimerisation on the function of human PD-1-CD28 IFP variants. To our surprise, length in relation to the physiological extracellular PD-1 domain did not result in a weaker effect as expected [26] but instead turned out to be fundamental for proper IFP function. Excess length resulted in the ability of the IFP to act as a CAR-independent T cell recognition and activation driver, requiring only the IFP ligand for its induction. We observed CAR-independent PD-L1-directed activation, proliferation, and tumour lysis in all T cells equipped with one of these IFP constructs (CTM + 12EC, CTM + 39EC, and CTM + 41EC) in vitro. We analyzed the properties of the shortest of these constructs (CTM + 12EC) in a xenograft in vivo model with critical results: T cells transduced with the CTM + 12EC IFP induced toxicities in all mice receiving this treatment (regardless of combination with an anti-CD19 CAR or not) and led to premature termination of the experiment within this treatment group, even before the development of a tumour signal. While we have not tested the even longer CTM + 39EC and CTM + 41EC IFP constructs in vivo, their potent PD-L1-directed and CAR-independent effect has been shown throughout all in vitro experiments. This property itself already deters from further therapeutic consideration and urges thorough evaluation of similar designer IFPs for clinical use.

Regarding the IFP constructs with physiological extracellular PD-1 length (PTM, CTM, and CTMΔ12EC), we could confirm that they augment CAR T therapy by overcoming PD-1-mediated suppression, in vitro and in vivo [19, 22–25, 52] without inducing toxicity or CAR-independent activation. However, in our model, we found that the propensity of the CTMΔ12EC IFP to dimerise through a disulphide linkage was dispensable for IFP activity and neither added to its function nor selectivity. In fact, T cells transduced with an anti-CD19 CAR and the CTMΔ12EC IFP mainly yielded similar results in vitro compared to the combination of the CAR with PTM or CTM, respectively. The CTMΔ12EC IFP tended to induce the highest IFN-γ, IL-2, and Granzyme B secretion out of all IFP constructs in combination with the anti-CD19 CAR in vitro but did not impact the therapeutic outcome in vivo.

In summary, our results demonstrate that human PD-1-based IFPs come with needs of their own: In our experiments, the addition of dimerisation-prone motifs did not increase the functionality of the IFP. We instead found that physiological extracellular PD-1 length was essential for its selective function and therapeutic application. It remains to be seen if this length requirement transfers to other IFP families and challenges previous models. As the race is still on to determine the optimal IFP design, bench-to-bedside translational studies must be aware of the effects that different structural choices might entail and adapt accordingly.

## Supplementary information

Supplementary Figures

## Data Availability

All data supporting this study are included in this published article and its Supplementary files. Raw datasets will be made available from the corresponding author upon reasonable request.
