# Supplementary material for: Rational design of PD-1-CD28 immunostimulatory fusion proteins for CAR T cell therapy
Source: Br J Cancer. 2023 Jul 4;129(4):696–705. doi: 10.1038/s41416-023-02332-9 (PMC10421897; doi:10.1038/s41416-023-02332-9)
Supplement: Supplementary file 1 — Supplementary Figures [file 41416_2023_2332_MOESM1_ESM.pdf]

Suplementary Figure 1

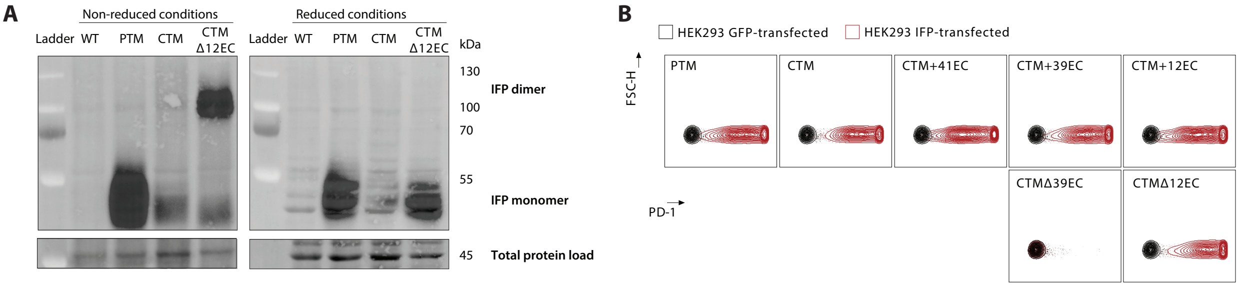

A Blot of monomeric or dimeric state of IFP constructs with physiological extracellular length (PTM, CTM and CTMΔ12EC) on transduced or wild type (WT) Jurkat cells. SDS-PAGE reveals dimeric state of CTMΔ12EC in non-reduced condition, which is reverted to a single band under reducing conditions. Depicted image is representative of 3 independently performed experiments. **B** Flow cytometry analysis of IFP expression (determined through PD-1 antibody staining) on HEK293 cells after plasmid transfection with different IFP variants or GFP as control. Experiment depicted is representative of 3 independent assays.

Supplementary Figure 2

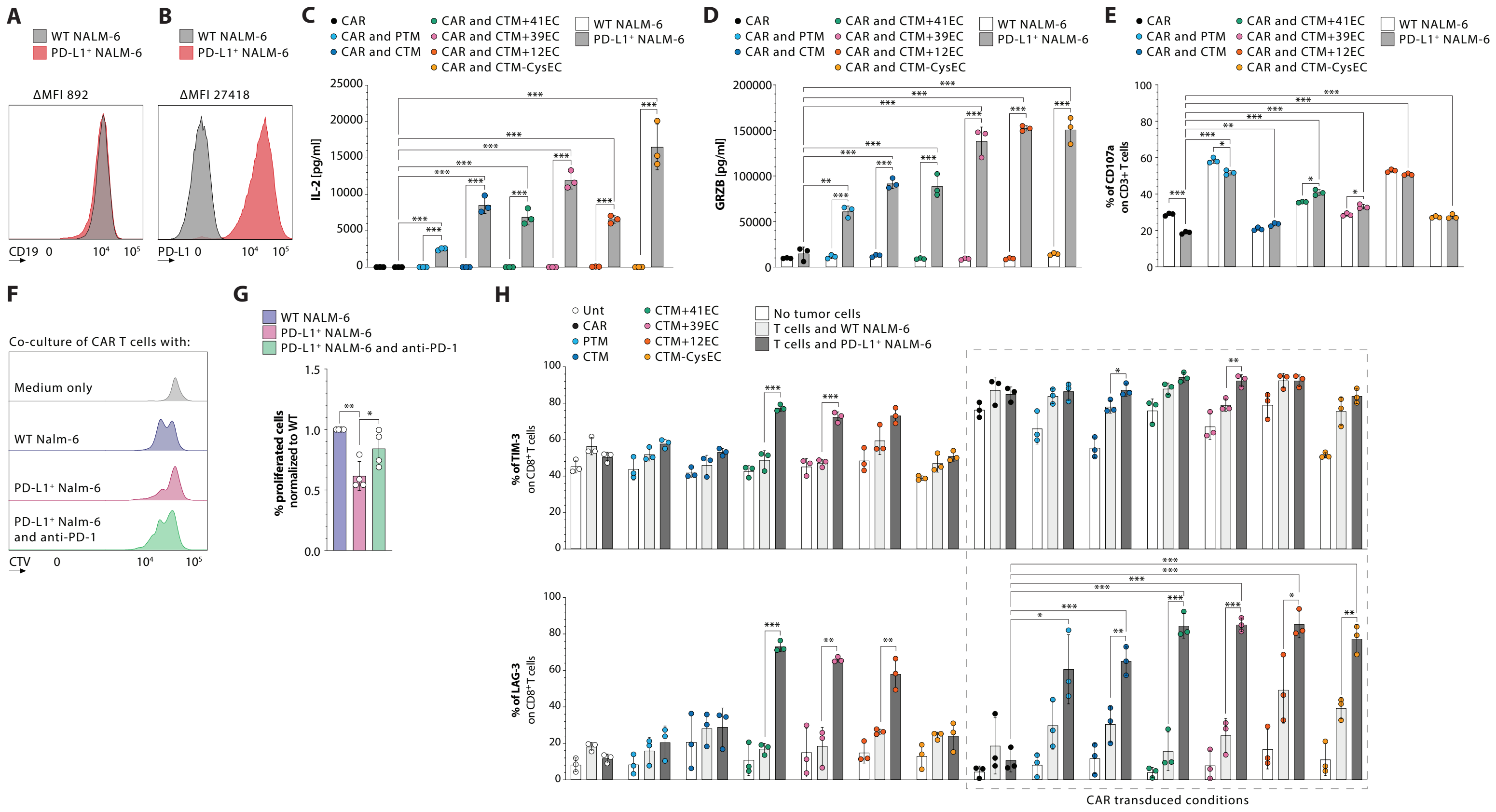

A Representative flow cytometry analysis of target antigen expression levels on Nalm-6 tumor cells for CD19 and **B** PD-L1. Staining for target antigens was carried out twice, one representative experiment is depicted. **C** and **D** Results of ELISA for IL-2 and Granzyme B concentration in the coculture supernatant. Supernatants were harvested from a coculture assay with Nalm-6 tumor cells after 24 hours (Granzyme B) and 48 hours (IL-2). Data is representative of 3 independent experiments with different donors. **E** Flow cytometry analysis of CD107a expression on transduced T cells after 4-6 hours of coculture with NALM-6 tumor cells. Data is representative of 3 independent experiments with different donors. **F** and **G** Flow cytometric readout of CTV-labeled and proliferated anti-CD19 CAR T cells after 72 hours of coculture with Nalm-6 cells and addition of an anti-PD-1 antibody (10 µg/ml) 30 minutes before coculture. Histograms in **F** are representative of one donor. Pooled data from all donors is depicted in **G**. **H** Flow cytometry analysis of TIM-3 and LAG-3 (depicted as % of positive cells on CD8+ T cells) on T cells after coculture experiments with NALM-6 tumor cells. Data pooled from independent experiments with different donors. P values are based on a two-tailed unpaired t-test.
